# Impact of interventionalist’s experience and gender on radiation dose and procedural time in CT-guided interventions—a retrospective analysis of 4380 cases over 10 years

**DOI:** 10.1007/s00330-020-07185-x

**Published:** 2020-08-26

**Authors:** Dorothea Theilig, Anna Mayerhofer, David Petschelt, Aboelyazid Elkilany, Bernd Hamm, Bernhard Gebauer, Dominik Geisel

**Affiliations:** Charité – Universitätsmedizin Berlin, corporate member of Freie Universität Berlin, Humboldt-Universität zu Berlin, Department of Diagnostic and Interventional Radiology, Augustenburger Platz 1, 13353 Berlin, Germany

**Keywords:** Tomography x-ray computed, Radiology interventional, Risk factors

## Abstract

**Objectives:**

To investigate the impact of the interventionalist’s experience and gender on radiation dose and procedural time in CT-guided interventions.

**Methods:**

We retrospectively analyzed 4380 CT-guided interventions performed at our institution with the same CT scanner from 2009 until 2018, 1287 (29%) by female and 3093 (71%) by male interventionalists. Radiation dose, number of CT fluoroscopy images taken per intervention, total procedural time, type of intervention, and degree of difficulty were derived from the saved dose reports and images. All 16 interventionalists included in this analysis performed their first CT-guided interventions during the study period, and interventions performed by each interventionalist were counted to assess the level of experience for each intervention in terms of the number of prior interventions performed by her or him. The Mann-Whitney *U* test (MWU test), multivariate regression, and linear mixed model analysis were performed.

**Results:**

Assessment of the impact of gender with the MWU test revealed that female interventionalists took a significantly smaller number of images (*p* < 0.0001) and achieved a lower dose-length product per intervention (*p* < 0.0001) while taking more time per intervention (*p* = 0.0001). This finding was confirmed for most types of interventions when additionally accounting for other possible impact factors in multivariate regression analysis. In linear mixed model analysis, we found that radiation dose, number of images taken per intervention, and procedural time decreased statistically significantly with interventionalist’s experience.

**Conclusions:**

Radiation doses of CT-guided interventions are reduced by interventionalist’s experience and, for most types of interventions, when performed by female interventionalists.

**Key Points:**

*• Radiation doses in CT-guided interventions are lower when performed by female interventionalists.*

*• Procedural times of CT-guided interventions are longer when performed by female interventionalists.*

*• Radiation doses of CT-guided interventions decrease with the interventionalist’s experience.*

**Electronic supplementary material:**

The online version of this article (10.1007/s00330-020-07185-x) contains supplementary material, which is available to authorized users.

## Introduction

Computed tomography (CT)-guided interventions allow accessing specific structures throughout the body precisely while posing comparatively little risks. Compared to open surgery, CT-guided interventions are far less invasive and require less anesthesia, resulting in lower health care costs [1]. The advent of CT fluoroscopy further improved interventional procedures [2, 3]. For these reasons, CT-guided interventions play an increasingly important role in routine clinical care today.

Radiation exposure of patients, interventionalists, and other medical staff present in the room remains one of the greatest concerns with CT-guided interventions. From all the different applications of ionizing radiation, CT-guided interventions are considered to come along with the greatest radiation exposure for interventional radiologists [4, 5]. In Germany, medical staff in areas with potential radiation exposure is legally required to wear personal dosimeters in order to estimate the radiation dose deposited as occupational radiation exposure must not exceed certain thresholds [6]. However, it is not only of interest for medical personnel to know whether they reached certain radiation thresholds as evaluated with personal dosimeters but also to have some knowledge about relative radiation exposure associated with certain procedures. This knowledge might help to reduce unjustified worries and at the same time sharpen awareness for potential dangers [7, 8].

The automatically generated dose reports provide excellent means to retrospectively compare the amounts of radiation applied per intervention. Although the dose report provides the radiation exposure of the patient and not that of the medical personnel present during the intervention, patient exposure can be used as a surrogate parameter for staff exposure as these two can be assumed to correlate with each other [9–12]. Nevertheless, there are other factors that have an impact on the amount of radiation medical staff is actually subjected to; these include the amount of lead protection used and the distance and angle of the personnel to the CT tube [13].

The observation that applied radiation dose varies considerably at our institution even between very similar interventions prompted us to investigate the mean radiation dose per intervention type and to identify factors that affect it. The actual dose is proportional to the number of CT fluoroscopy images taken and the amount of milliampere-seconds and kilovolts used with a higher milliampere-second output improving spatial resolution of the CT images at the cost of a higher radiation dose [14–17]. The focus was on the level of experience, and we hypothesize that the applied amount of ionizing radiation decreases with the interventionalist’s experience. Another factor of interest was the interventionalist’s gender with the hypothesis that women of childbearing age tend to be more concerned about minimizing radiation exposure compared to their male counterparts. It must be pointed out, though, that there are other factors that could be hypothesized to affect the interventionalist’s behavior with regard to application of radiation during interventional procedures like documentation against possible future medical malpractice claims.

## Materials and methods

### Study design

The study was approved by the local ethics committee. The ethics committee waived informed consent requirements for this retrospective study.

We retrospectively analyzed 4468 interventions of 5 different types that were performed at our institution with the same CT scanner from 2009 through 2018. During that period, a total of 16 residents (4 women and 12 men) started their training in CT-guided interventions and were included in the analysis. All interventions of the 5 different types performed during that time period by one of these interventionalists were initially included, and 88 interventions that were either not completed or had incomplete documentation were subsequently excluded again leaving 4380 interventions for the final analysis.

Data were retrieved from the dose reports automatically generated for each intervention and CT fluoroscopy images, both stored in the PACS, and comprised milliampere-seconds, kilovolts, dose-length product (DLP), time taken for the intervention counted in minutes from the timepoint of the first to the last CT fluoroscopy image taken as recorded on the image (procedural time), and whether it was an in-plane or out-of-plane puncture with the latter being more difficult (see standard approach to intervention types in supplementary material). For periradicular therapies (PRTs), it was also noted whether the localization was cervical or lumbar as cervical PRTs are considered to be more difficult than lumbar PRTs due to the close proximity of delicate structures like the vertebral arteries and spinal cord.

All data were collected for each interventionalist separately counting the number of interventions in chronological order as recorded in the radiological information system (RIS). Intervention-specific experience of the interventionalist was defined as the number of interventions of the same type performed by that interventionalist up to the time of the intervention in question. General experience of the interventionalist was defined as the number of all interventions performed by that interventionalist up to the time of the intervention in question.

### CT intervention

All CT-guided interventions were performed with the same CT scanner, a Siemens Definition AS with a 32-row detector and a z-flying focal spot. Either the so-called quick-check technique with intermittent 5-mm single-slice images taken or a combination of the quick-check technique and continuous CT fluoroscopy was used. The quick-check technique is essentially analogous to conventional CT except for faster reconstruction times and manual table positioning by the radiologist [5].

The CT scanner was operated from inside the scanning room via a foot pedal. Two monitors were positioned next to the patient on the opposite side to the interventionalist. One monitor was generally used to view the planning scan or prior contrast-enhanced scans during the intervention, while the other monitor displayed the CT fluoroscopy scans in real time. A joystick panel attached to the stationary part of the CT scanner was used to change the CT table position as well as the images and measurements displayed on the monitors.

### Types of interventions and interventional workflow

Five different types of interventions regularly performed by the radiologists of our department were deemed most suitable for our purpose because they are highly comparable and remained so over the 10-year study period:

1. Periradicular therapy (PRT)
2. Liver biopsy
3. Lung biopsy
4. Drain insertion into abdominal fluid collection (abdominal drain)
5. Drain insertion into pleural fluid collection (chest drain)

Except for PRT, a conventional CT scan of the target region obtained prior to the intervention without medical staff present in the scanning room was used to plan the best way to access the lesion. For PRT, a sagittal scout was used to plan a 5-mm single-slice CT image of the target region, which was repeated in a slightly different position if necessary. As with conventional CT scans in the other interventions, this image was used for puncture planning. Conventional planning CT scans (helical or single slices) were not included in the analysis.

For each of the five types of interventions included in our analysis, a standardized preset of milliampere-seconds and kilovolts was used for acquiring CT fluoroscopy images unless the interventionalist deemed it necessary to alter it. The standardized presets per intervention type, which remained the same over the 10-year study period, were as follows: 20 mAS and 100 kV for periradicular therapy, 60 mAS and 100 kV for liver biopsy, 30 mAS and 120 kV for lung biopsy, 60 mAS and 120 kV for abdominal drains, and 30 mAS and 120 kV for chest drains. The standard approach to each intervention type is outlined in the supplementary material.

### Analysis and statistics

The Shapiro-Francia *W*′ test and the skewness and kurtosis test were used to test for normal distribution. The Mann-Whitney *U* test (MWU test) was used as a nonparametric test to compare continuous variables between two groups.

Multivariate regression analysis was performed to analyze the impact of the interventionalist’s gender on the output parameters (DLP, number of fluoroscopy images taken per intervention, and procedural time) while also accounting for other influencing factors.

To determine the impact of experience on the output parameters, we performed linear mixed model analysis of the chronologically numbered interventions of the same type per interventionalist with gender and parameters reflecting the degree of difficulty as covariates.

Aiming to identify the turning points in the learning curve of interventionalists, we divided the successively performed interventions by each interventionalist into clusters of 10 for each intervention type. Linear regression analysis with pairwise comparisons of margins was preformed between these clusters.

A *p* value of < 0.05 was considered statistically significant. All statistical analyses were performed with Stata/MP Version 16 (StataCorp).

## Results

The 4380 interventions analyzed included 1950 (44.52%) PRTs, 502 (11.46%) liver biopsies, 411 (9.38%) lung biopsies, 1020 (23.29%) abdominal drains, and 497 (11.35%) chest drains. Female interventionalists performed 1287 (29%) of the 4380 procedures, and male interventionalists, 3093 (71%). Descriptive statistics with the exact numbers of interventions performed by females and males are compiled in Table 1.

**Table 1:** Descriptive statistics of the 4280 CT-guided interventions included in our retrospective analysis. For categorical variables, the percentage (%) and for continuous variables the standard deviation (± SD) is given

Type of intervention			All	Female interventionalist	Male interventionalist
Periradicular therapy (PRT)	Number		1950	671	1279
	DLP (mGy*cm)		27.7 ± 41.6	11.4 ± 11.7	36.3 ± 48.5
	No. of images		22.1 ± 23.4	16.5 ± 10.0	25.1 ± 27.6
	Procedural time (min)		6.5 ± 4.4	6.9 ± 4.4	6.3 ± 4.4
	Localization	Cervical spine	461 (24%)	165 (25%)	299 (23%)
		Lumbar spine	1479 (76%)	506 (75%)	980 (77%)
Liver biopsy	Number		502	122	380
	DLP (mGy*cm)		130.9 ± 147.8	101.0 ± 93.8	140.5 ± 160.3
	No. of images		57.6 ± 52.7	47.1 ± 36.4	61.1 ± 56.6
	Procedural time (min)		13.3 ± 9.0	15.2 ± 10.2	12.7 ± 8.6
	Technique	In-plane	226 (45%)	80 (66%)	146 (38%)
		Out-of-plane	276 (55%)	42 (34%)	234 (62%)
Lung biopsy	Number		411	124	287
	DLP (mGy*cm)		59.0 ± 63.9	40.7 ± 25.5	66.8 ± 73.2
	No. of images		55.5 ± 50.6	43.1 ± 25.6	60.8 ± 57.4
	Procedural time (min)		12.9 ± 9.0	14.0 ± 7.2	12.5 ± 9.6
Abdominal drain	Number		1020	259	761
	DLP (mGy*cm)		90.4 (± 104.7)	86.0 (± 98.2)	91.9 (± 106.8)
	No. of images		42.6 (± 41.3)	41.7 (± 39.5)	42.9 (± 41.9)
	Procedural time (min)		11.7 (± 8.8)	13.2 (± 9.5)	11.1 (± 8.5)
	Technique	In-plane	468 (46%)	126 (49%)	342 (45%)
		Out-of-plane	552 (54%)	133 (51%)	419 (55%)
Chest drain	Number		497	111	386
	DLP (mGy*cm)		40.9 (± 40.6)	28.5 (± 23.1)	44.5 (± 43.7)
	Images		32.4 (± 23.1)	25.7 (± 15.8)	34.2 (± 24.4)
	Procedural time (min)		11.9 (± 8.0)	12.3 (± 6.7)	11.8 (± 8.3)

**Fig. 1:**
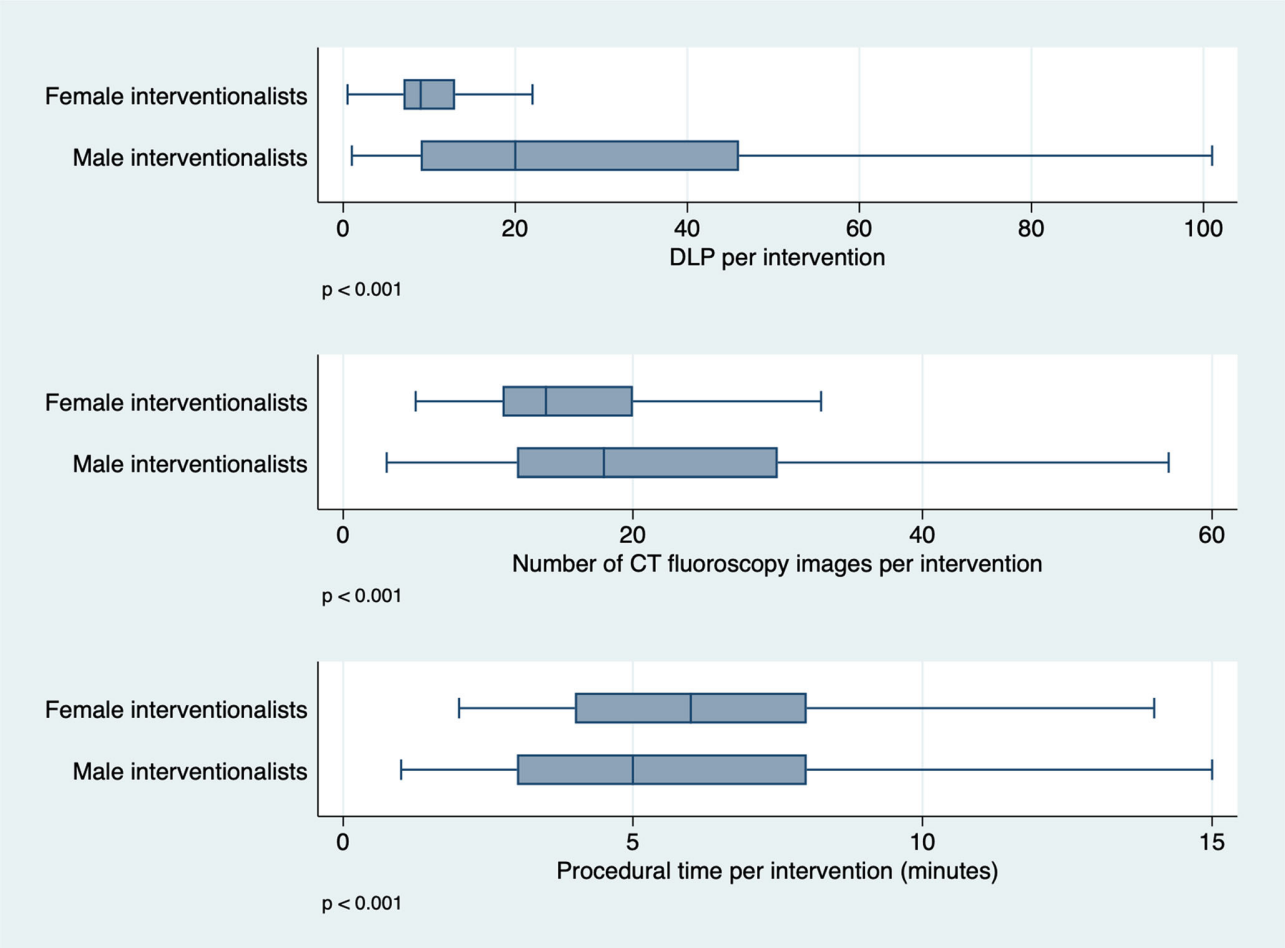
Boxplot diagram of DLP, number of fluoroscopy images taken, and procedural time for PRT interventions by female and male interventionalists

Analysis of gender-related differences with the MWU test revealed that female interventionalists took a statistically significantly smaller number of images (*p* < 0.0001) and achieved a significantly lower DLP per intervention (*p* < 0.0001) while taking significantly more time per intervention (*p* = 0.0001). Separate analysis of each of the five different types of interventions yielded similar results with the notable exception that, for abdominal drains, the DLP and number of images taken were similar for male and female interventionalists. Table 2 summarizes MWU test results for the different types of interventions, and Fig. 1 provides a boxplot diagram of the results for PRTs.

**Table 2:** Mann-Whitney *U* test comparing female versus male interventionalists with respect to DLP, number of images taken, and procedural time for the 5 types of CT-guided interventions analyzed

Type of intervention	Output parameter	*p* value	Sig.
Periradicular therapy (PRT)	DLP	< 0.0001	***
	Number of images	< 0.0001	***
	Procedural time	< 0.0001	***
Liver biopsy	DLP	0.0057	**
	Number of images	0.0182	*
	Procedural time	0.0113	*
Lung biopsy	DLP	0.0011	**
	Number of images	0.0121	**
	Procedural time	0.0003	***
Abdominal drain	DLP	0.4459	
	Number of images	0.7475	
	Time	< 0.0001	***
Chest drain	DLP	< 0.0001	***
	Number of images	0.0008	***
	Procedural time	0.0832	

*p < 0.05; **p < 0.01; ***p < 0.001

With multivariate regression analysis including other influencing factors, namely specific and general experience of the interventionalist as well as degree of difficulty of the intervention captured as in-plane versus out-of-plane puncture pathways for liver biopsy and abdominal drain insertion and cervical versus lumbar location for PRTs, we still found that female interventionalists achieved a statistically significantly lower DLP and took fewer images in PRTs (*p* < 0.001 each), lung biopsies (*p* < 0.001 and *p* = 0.003), and chest drains (*p* < 0.001 each) while taking statistically significantly longer for PRTs (*p* < 0.001) and liver biopsies (*p* = 0.007). The other impact factors analyzed also showed a statistically significant impact in the majority of cases, i.e., either specific or general experience had a significant impact on at least one of the three output parameters, location of PRT had a significant impact on procedural time, and out-of-plane puncture versus in-plane puncture had a significant impact on all three output parameters. Results of the multivariate regression analysis are summed up in Table 3.

**Table 3:** Multivariate regression analysis of the three output parameters DLP, number of images taken, and procedural time for the 5 types of interventions analyzed. *p* value of all models < 0.001

Type of intervention	Output parameter	Impact factors analyzed	Coef.	95% conf. interval		*p* value	Sig.
Periradicular therapy (PRT)	DLP	Specific experience	− 0.215	− 0.299	− 0.132	< 0.001	***
		General experience	0.002	0.024	0.038	0.922	
		Cervical (versus lumbar)	1.640	− 2.347	5.623	0.420	
		Female (versus male) interventionalist	− 20.399	− 24.301	− 16.497	< 0.001	***
	No. of images	Specific experience	− 0.0982	− 0.148	− 0.049	< 0.001	***
		General experience	0.000	− 0.021	0.021	0.995	
		Cervical (versus lumbar)	2.058	− 0.290	4.406	0.086	
		Female (versus male) interventionalist	− 6.542	− 8.841	− 4.243	< 0.001	***
	Procedural time	Specific experience	− 0.011	− 0.020	− 0.002	0.015	*
		General experience	− 0.007	− 0.011	− 0.003	< 0.001	***
		Cervical (versus lumbar)	0.585	0.157	1.012	0.007	**
		Female (versus male) interventionalist	0.895	0.476	1.314	< 0.001	***
Liver biopsy	DLP	Specific experience	0.230	− 2.149	2.609	0.849	
		General experience	− 0.209	− 0.510	0.093	0.174	
		Out-of-plane (versus in-plane)	98.280	75.176	121.384	< 0.001	***
		Female (versus male) interventionalist	− 2.876	− 34.844	29.093	0.860	
	No. of images	Specific experience	− 0.378	− 1.255	0.499	0.398	
		General experience	− 0.019	− 0.130	0.092	0.736	
		Out-of-plane (versus in-plane)	41.889	33.369	50.408	< 0.001	***
		Female (versus male) interventionalist	− 3.071	− 14.859	8.718	0.609	
	Procedural time	Specific experience	− 0.213	− 0.366	− 0.060	0.006	**
		General experience	0.005	− 0.014	0.025	0.585	*
		Out-of-plane (versus in-plane)	5.257	3.873	6.840	< 0.001	***
		Female (versus male) interventionalist	2.827	0.774	4.880	0.007	**
Lung biopsy	DLP	Specific experience	2.464	0.694	4.234	0.006	**
		General experience	− 0.326	− 0.498	− 0.154	< 0.001	***
		Female (versus male) interventionalist	− 23.630	− 36.629	− 10.632	< 0.001	***
	No. of images	Specific experience	1.769	0.357	3.180	0.014	*
		General experience	− 0.240	− 0.377	− 0.103	0.001	**
		Female (versus male) interventionalist	− 15.838	− 26.203	− 5.472	0.003	**
	Procedural time	Specific experience	− 0.025	− 0.273	0.224	− 0.845	
		General experience	− 0.016	− 0.040	0.008	0.198	
		Female (versus male) interventionalist	1.523	− 0.301	3.348	0.102	
Abdominal drain	DLP	Specific experience	0.027	− 0.245	0.299	0.844	
		General experience	− 0.155	− 0.249	− 0.061	0.001	**
		Out-of-plane (versus in-plane)	55.841	43.838	67.844	< 0.001	***
		Female (versus male) interventionalist	− 12.544	− 26.862	1.774	0.086	
	No. of images	Specific experience	− 0.049	− 0.154	0.057	0.365	
		General experience	− 0.040	− 0.076	− 0.003	0.033	*
		Out-of-plane (versus in-plane)	26.233	21.571	30.896	< 0.001	***
		Female (versus male) interventionalist	− 4.337	− 9.898	1.225	0.126	
	Procedural time	Specific experience	− 0.001	− 0.024	0.021	0.911	
		General experience	− 0.016	− 0.024	− 0.008	< 0.001	***
		Out-of-plane (versus in-plane)	3.243	2.253	4.233	< 0.001	***
		Female (versus male) interventionalist	1.171	− 0.009	2.352	0.052	
Chest drain	DLP	Specific experience	− 0.371	− 0.913	0.172	0.180	
		General experience	− 0.013	− 0.087	0.061	0.729	
		Female (versus male) interventionalist	− 20.581	− 29.682	− 11.480	< 0.001	***
	No. of images	Specific experience	− 0.277	− 0.580	0.026	0.073	
		General experience	− 0.006	− 0.047	0.035	0.774	
		Female (versus male) interventionalist	− 11.792	− 16.875	− 6.709	< 0.001	***
	Procedural time	Specific experience	− 0.156	− 0.260	− 0.052	0.003	**
		General experience	0.004	− 0.010	0.018	0.578	
		Female (versus male) interventionalist	− 1.099	− 2.842	0.644	0.216	

Specific experience, number of interventions of the same type performed by the interventionalist before the intervention in question. General experience, number of all interventions by performed by the interventionalist before the intervention in question

*p < 0.05; **p < 0.01; ***p < 0.001

**Fig. 2:**
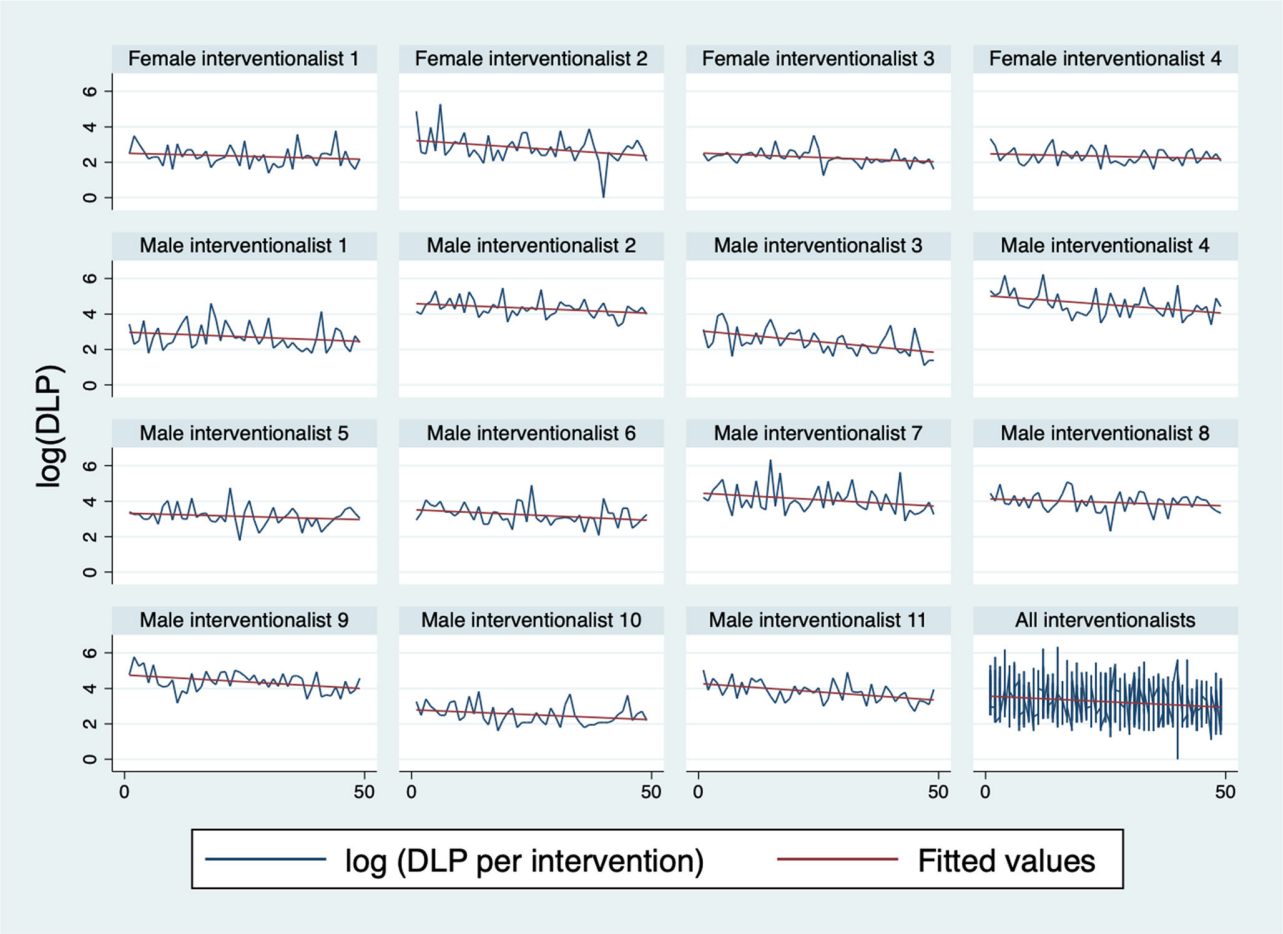
Graph depicting DLP over successively performed PRT procedures for each interventionalist analyzed

In linear mixed model analysis of the chronologically numbered interventions of the same type per interventionalist with parameters reflecting the degree of difficulty and gender as covariates, we found that the number of interventions of the same type performed prior to the one in question had a significant negative impact on the output parameters, i.e., the more experienced the interventionalist, the lower the DLP, the fewer the images taken, and the shorter the procedure. All results of the linear mixed model analysis are provided in Table 4. Figure 2 depicts DLP per PRT over successively performed PRT interventions for each interventionalist analyzed.

**Table 4:** Linear mixed model analysis of the three output parameters DLP, number of images taken, and procedural time for the 5 types of interventions analyzed

Type of intervention	Output parameter		Coef.	95% conf. interval		*p* value	Sig.
Periradicular therapy (PRT)	DLP	No. of interventions	− 0.155	− 0.186	− 0.124	< 0.001	***
		Cervical (versus lumbar)	2.168	− 1.311	5.646	0.222	
		Female (versus male) interventionalist	− 24.543	− 49.481	0.395	0.054	
	No. of images	No. of interventions	− 0.078	− 0.098	− 0.059	< 0.001	***
		Cervical (versus lumbar)	2.037	− 0.191	4.265	0.073	
		Female (versus male) interventionalist	− 7.87	− 18.017	2.267	0.128	
	Procedural time	No. of interventions	− 0.0236	− 0.027	− 0.012	< 0.001	***
		Cervical (versus lumbar)	0.651	0.244	1.057	0.002	**
		Female (versus male) interventionalist	0.826	− 0.761	2.414	0.308	
Liver biopsy	DLP	No. of interventions	− 1.331	− 2.133	− 0.530	0.001	**
		Out-of-plane (versus in-plane)	91.346	67.460	115.231	< 0.001	***
		Female (versus male) interventionalist	− 21.130	− 83.737	41.478	0.508	
	No. of images	No. of interventions	− 0.459	− 0.739	− 0.178	0.001	**
		Out-of-plane (versus in-plane)	40.045	31.540	48.551	< 0.001	***
		Female (versus male) interventionalist	− 5.714	− 23.394	11.966	0.526	
	Procedural time	No. of intervention	− 0.111	0.024	− 0.159	− 0.064	*
		Out-of-plane (versus in-plane)	6.065	4.645	7.485	< 0.001	***
		Female (versus male) interventionalist	3.046	− 0.959	7.052	0.136	
Lung biopsy	DLP	No. of interventions	− 0.533	− 1.008	− 0.059	0.028	*
		Female (versus male) interventionalist	− 28.79	− 62.751	5.177	0.097	
	No. of images	No. of interventions	− 0.424	− 0.803	− 0.450	0.028	*
		Female (versus male) interventionalist	− 19.661	− 45.312	5.991	0.133	
	Procedural time	No. of interventions	− 0.119	− 0.186	− 0.053	< 0.001	***
		Female (versus male) interventionalist	1.016	− 3.104	5.136	0.629	
Abdominal drain	DLP	No. of interventions	− 0.290	− 0.411	− 0.168	< 0.001	***
		Out-of-plane (versus in-plane)	54.089	42.402	65.775	< 0.001	***
		Female (versus male) interventionalist	− 25.299	− 69.811	19.213	0.265	
	No. of images	No. of interventions	− 0.121	− 0.168	− 0.074	< 0.001	***
		Out-of-plane (versus in-plane)	25.766	21.165	30.367	< 0.001	***
		Female (versus male) interventionalist	− 8.974	− 22.301	4.353	0.187	
	Procedural time	No. of interventions	− 0.030	− 0.040	− 0.020	< 0.001	***
		Out-of-plane (versus in-plane)	3.255	2.289	4.221	< 0.001	***
		Female (versus male) interventionalist	0.194	− 3.141	3.529	0.909	
Chest drain	DLP	No. of interventions	− 0.251	− 0.428	− 0.075	0.005	**
		Female (versus male) interventionalist	− 21.906	− 42.506	− 1.306	0.037	*
	No. of images	No. of interventions	− 0.194	− 0.292	− 0.096	< 0.001	***
		Female (versus male) interventionalist	− 12.863	− 23.306	− 2.421	0.016	*
	Procedural time	No. of interventions	− 0.091	− 0.125	− 0.058	< 0.001	***
		Female (versus male) interventionalist	− 1.308	− 3.842	1.227	0.312	

No. of interventions, number of interventions of the same type by the same interventionalist

*p < 0.05; **p < 0.01; ***p < 0.001

**Fig. 3:**
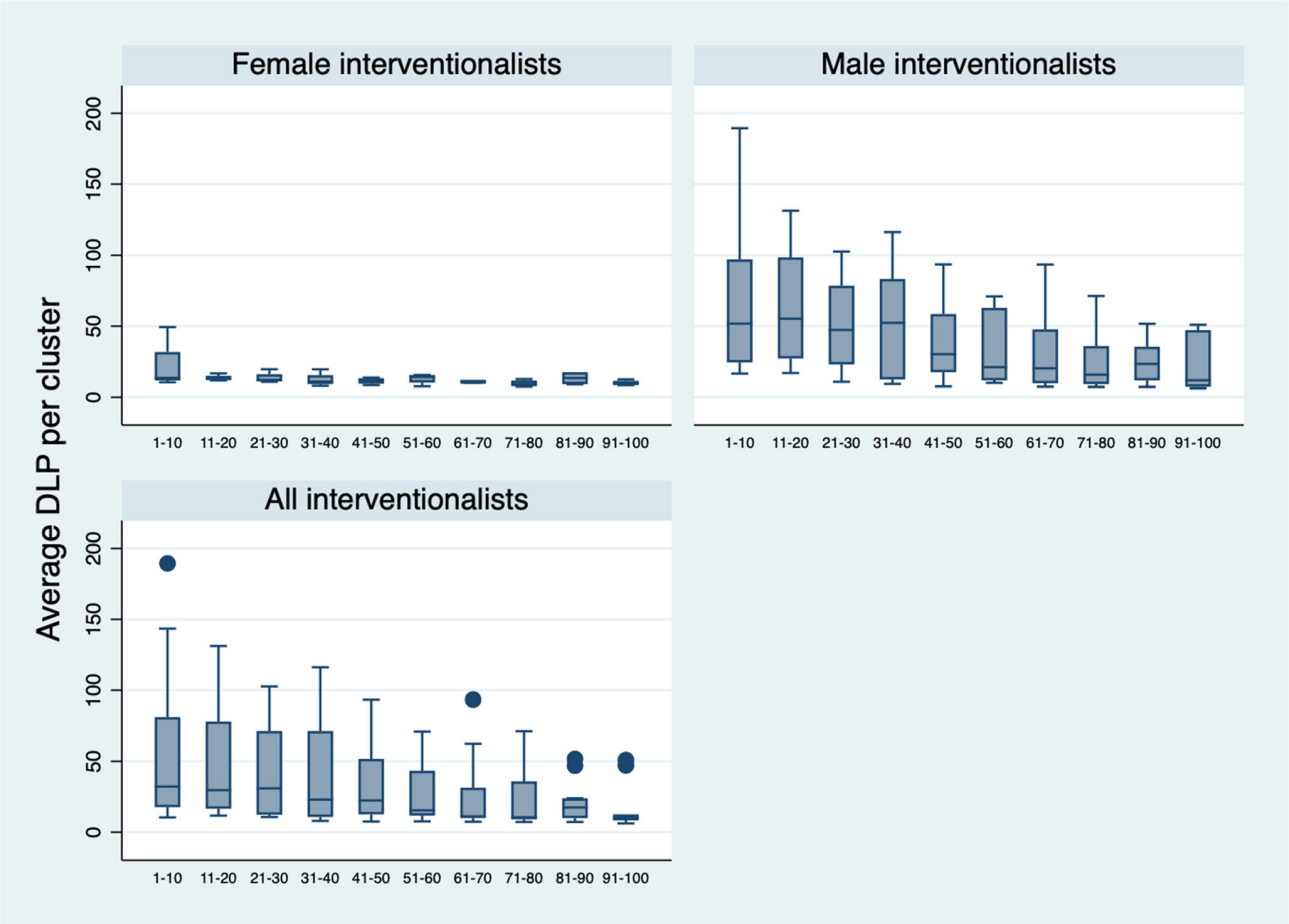
Boxplot diagram of DLPs of PRT procedures successively performed by each interventionalist in clusters of 10 displayed for female, male, and all interventionalists

When trying to evaluate the learning curve of the interventionalist, the PRT subgroup is most suitable because it is the largest group with each interventionalist having performed sufficient interventions of the type. We divided the first successively performed 100 PRTs by each interventionalist into clusters of 10, and the resulting 10 clusters are depicted in Fig. 3 as boxplot diagrams for female, male, and all interventionalists together. In linear regression analysis with pairwise comparisons of these clusters, we found that for all interventionalists taken together, the cluster of the 41th to 50th PRT was the first to differ significantly (*p* = 0.032) from the first cluster, i.e., 1st to 10th PRT performed. For abdominal drains, the 31th to 40th intervention was the first cluster to differ significantly (*p* = 0.016) from the 1st to 10th intervention. For liver biopsy, lung biopsy, and chest drains, no statistically significant difference was found between the clusters. Visualization of the clusters for these types of interventions are provided in Figure I–IV and results of linear regression analysis with pairwise comparisons for all types of interventions are provided in Table I–V of the supplementary material.

**Fig. 4:**
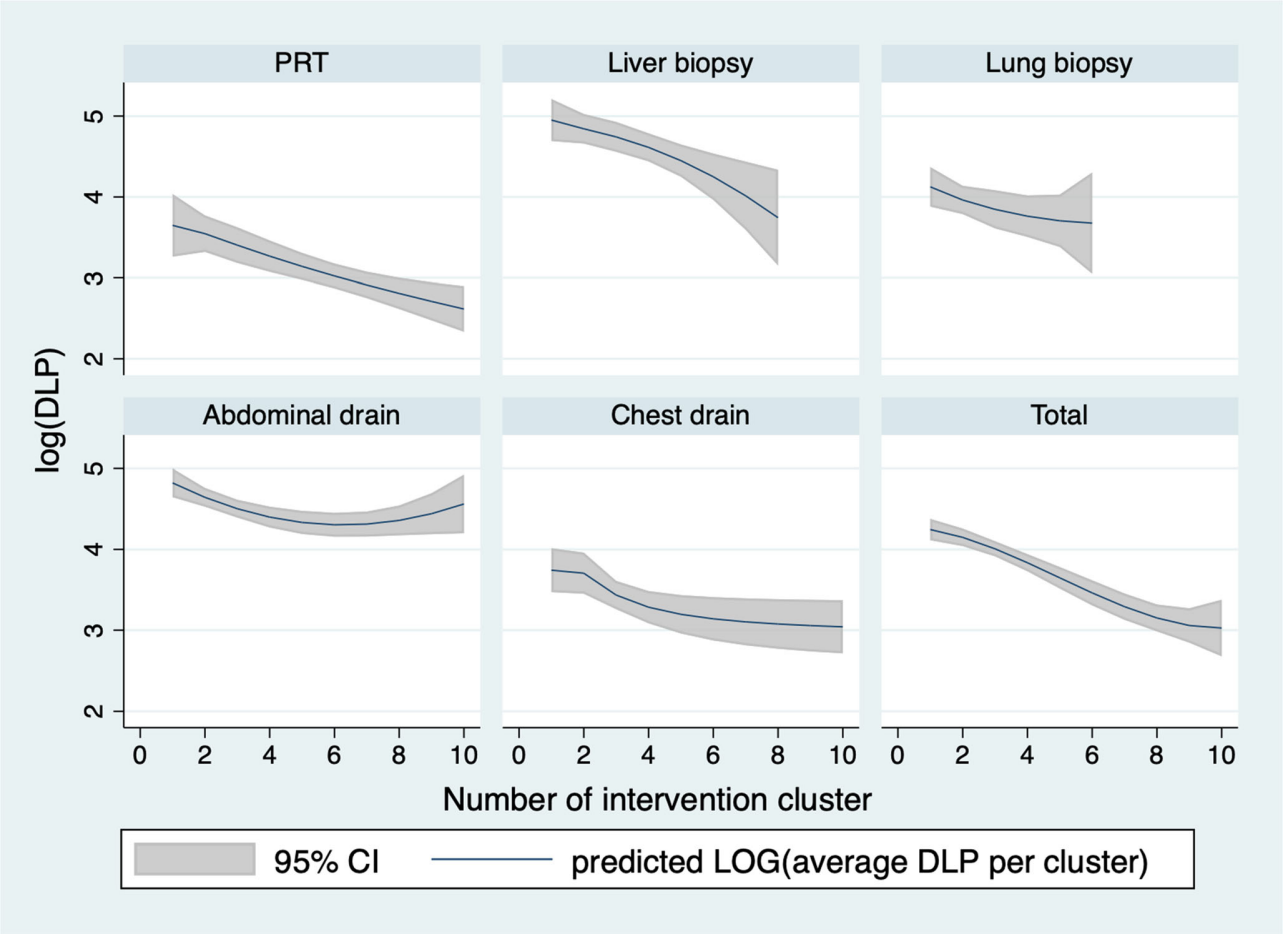
Graph of average DLPs with 95% confidence intervals of interventions successively performed by each interventionalist in clusters of 10 depicted over the number of interventions for each intervention type

Figure 4 depicts average DLPs per intervention with respect to successive intervention clusters for all five types of interventions analyzed in this study. It illustrates that with all types of interventions except for abdominal drains, DLP decreased between consecutively performed interventions, i.e., experience.

## Discussion

Our finding that procedure time decreases with the number of previously performed CT-guided interventions, i.e., experience, as unambiguously proven in this study, was somewhat expected and is in accordance with the results of similar studies in related and other fields such as fluoroscopy-guided facet joint injections [18], fluoroscopically guided lumbar puncture [19], uterine artery embolization [20], mechanical thrombectomy in acute ischemic stroke [21], or laparoscopic colorectal surgery and gastrectomy [22, 23].

Procedure time is crucial in mechanical thrombectomy for acute ischemic stroke but not as important in CT-guided interventions, where radiation exposure is of greater concern instead. Our study shows that radiation exposure also decreases as the interventionalist’s experience increases, which is in accordance with the above-quoted study analyzing radiation exposure and experience in mechanical thrombectomy for acute ischemic stroke [21].

The findings of the analysis of successively performed interventions of the same type indicate that an interventionalist can be considered to have gained a relevant amount of experience after about 50 PRTs or 40 abdominal drains. For liver biopsy, lung biopsy, and chest drains, no such statistically significant difference was found between the clusters. This is most likely due to the smaller number of interventions of these intervention types and also possibly indicates a less steep learning curve.

Interestingly, we found that in most of the five CT-guided intervention types we analyzed, female interventionalists needed significantly fewer images and thus achieved lower DLPs compared to their male counterparts, a finding that, to our knowledge, has not been reported before. This supports the hypothesis that women of childbearing age are more concerned about radiation and thus make a greater effort to minimize occupational radiation exposure in order to minimize gonadal radiation exposure in view of possible future pregnancies. The striking differences in radiation doses for PRTs, lung biopsies, and chest drain procedures we observed between female and male interventionalists with, for example, female interventionalists accomplishing PRTs with approx. one third the DLP of male interventionalists, while there was no statistically significant gender-related radiation dose difference for liver biopsies and abdominal drain procedures in multiple regression analysis, might indicate that the latter two types of interventions leave less room to minimize radiation doses. It has to be pointed out, though, that the standard deviation was decidedly bigger between male interventionalists than between female interventionalists (see Fig. 3), which is probably at least in part due to the fact that more men were analyzed than women. Additionally, this might reflect the fact that there are some men who are just as concerned as women of childbearing age about radiation exposure while there are only some who are not.

On the other hand, female interventionalists needed statistically significantly longer for PRTs and liver biopsies in multivariate regression analysis. This might be due to other time-consuming precautions taken by female interventionalists to minimize radiation exposure not captured in DLP, for example, positioning oneself next to the CT tube during radiation application whenever possible. While statistically significant, the difference in average procedure time of 0.6 min for PRTs and 2.5 min for liver biopsies is small. This holds particularly true for CT-guided interventions, where time is less crucial than in other procedures like thrombectomy for cerebral embolism, as mentioned above.

The study has several limitations that need to be discussed, in particular the retrospective design and the fact that there was no equal gender distribution, with approximately two thirds of the study procedures being performed by male interventionalists and one third by female interventionists.

The study provides no information about the absolute radiation exposure of the medical personnel involved, which can only be obtained by directly measuring radiation exposure with dosimeters worn by the interventionalist, as done in a number of smaller studies [5, 24–27]. In this study, we used the patient dose as a surrogate parameter for staff dose and, while there are several studies showing the interdependence of patient and staff dose during radiologic interventions [9–12], we have not proven this for our setting, which therefore constitutes a limitation.

There are a couple of uncontrolled factors that might have affected the output parameters: The interventionalist is part of a team including a radiographer, nurse, and anesthetist. The experience and skills of these team members also affect procedural time and possibly even the number of CT images required. These team members change constantly, which is why we could not account for this variable in our retrospective study.

Furthermore, patients differ in how well they tolerate a CT-guided intervention. For example, overly anxious, pain-sensitive, or cognitively impaired patients can make it more difficult for the interventionalist by moving during the procedure. Other patient-related factors are size and weight. In obese patients, a higher radiation dose is necessary to achieve the same image quality, and the distance to the target lesion is longer.

Moreover, we must be aware that interventionalists might have also gained additional experience from other interventions performed during their training, which were not included in this study because they are less frequent, more case dependent, and less standardized, hampering direct comparison.

Another aspect difficult to account for in this retrospective setting is the degree of interaction between the resident in training and the experienced interventionalist. During the first couple of interventions performed by a new trainee, a senior interventionalist might have directly assisted or even completed the intervention if necessary without this being documented.

Although risks associated with CT-guided interventions are generally low, they are nevertheless present [28]. While the actual rate of complications is an interesting topic in itself and has been nicely described elsewhere [29], it was not the subject of this study.

In conclusion, our study shows that radiation exposure during CT-guided interventions decreases with the interventionalist’s experience and, for most types of interventions, is lower when the interventionalist is a woman.

## Electronic supplementary material

ESM 1 (DOCX 36243 kb)

## Abbreviations and acronyms

CT: Computed tomography
DLP: Dose-length product
kV: Kilovolt
mAs: Milliampere-seconds
mSv: Millisievert
MWU test: Mann-Whitney *U* test
PACS: Picture archiving and storage system
PRT: Periradicular therapy

## References

[1] Silverman SG, Deuson TE, Kane N (1998). Percutaneous abdominal biopsy: cost-identification analysis. Radiology.

[2] Carlson SK, Bender CE, Classic KL (2001). Benefits and safety of CT fluoroscopy in interventional radiologic procedures. Radiology.

[3] Leng S, Christner JA, Carlson SK (2011). Radiation dose levels for interventional CT procedures. AJR Am J Roentgenol.

[4] Alves JG, Sarmento S, Pereira JS (2019). Dose to the interventional radiologist in CTF-guided procedures. Radiat Environ Biophys.

[5] Paulson EK, Sheafor DH, Enterline DS, McAdams HP, Yoshizumi TT (2001). CT fluoroscopy--guided interventional procedures: techniques and radiation dose to radiologists. Radiology.

[6] German federal radiation protection act §78 I S.1 StrlSchG. Available via https://www.gesetze-im-internet.de/strlschg/__78.html. Accessed 30 Jun 2020

[7] Sadigh G, Khan R, Kassin MT, Applegate KE (2014). Radiation safety knowledge and perceptions among residents: a potential improvement opportunity for graduate medical education in the United States. Acad Radiol.

[8] Heyer CM, Peters S, Lemburg S, Nicolas V (2007). Awareness of radiation exposure of thoracic CT scans and conventional radiographs: what do non-radiologists know?. Rofo.

[9] Williams JR (1997). The interdependence of staff and patient doses in interventional radiology. Br J Radiol.

[10] Ciraj-Bjelac O, Antic V, Selakovic J, Bozovic P, Arandjic D, Pavlovic S (2016). Eye lens exposure to medical staff performing electrophysiology procedures: dose assessment and correlation to patient dose. Radiat Prot Dosimetry.

[11] Mohapatra A, Greenberg RK, Mastracci TM, Eagleton MJ, Thornsberry B (2013). Radiation exposure to operating room personnel and patients during endovascular procedures. J Vasc Surg.

[12] Vano E, Gonzalez L, Guibelalde E, Fernandez JM, Ten JI (1998). Radiation exposure to medical staff in interventional and cardiac radiology. Br J Radiol.

[13] Körner M, Linsenmaier U, Reiser MF, Wirth S (2010) Strategies for the reduction of exposure of medical staff in the CT operating room. Presented at European Congress of Radiology 2010, Vienna, Austria

[14] Lucey BC, Varghese JC, Hochberg A, Blake MA, Soto JA (2007). CT-guided intervention with low radiation dose: feasibility and experience. AJR Am J Roentgenol.

[15] Lamba R (2014). Radiation dose optimization for CT-guided interventional procedures in the abdomen and pelvis. J Am Coll Radiol.

[16] Sarti M, Brehmer WP, Gay SB (2012). Low-dose techniques in CT-guided interventions. Radiographics.

[17] Raman SP, Mahesh M, Blasko RV, Fishman EK (2013). CT scan parameters and radiation dose: practical advice for radiologists. J Am Coll Radiol.

[18] Dias TR, Alves Junior J, Abdala N (2017). Learning curve of radiology residents during training in fluoroscopy-guided facet joint injections. Radiol Bras.

[19] Faulkner AR, Bourgeois AC, Bradley YC, Hudson KB, Heidel RE, Pasciak AS (2015). Simulation-based educational curriculum for fluoroscopically guided lumbar puncture improves operator confidence and reduces patient dose. Acad Radiol.

[20] Das R, Lucatelli P, Wang H, Belli AM (2015). Identifying the learning curve for uterine artery embolisation in an Interventional Radiological Training Unit. Cardiovasc Intervent Radiol.

[21] Weyland CS, Hemmerich F, Mohlenbruch MA, Bendszus M, Pfaff JAR (2020). Radiation exposure and fluoroscopy time in mechanical thrombectomy of anterior circulation ischemic stroke depending on the interventionalist’s experience-a retrospective single center experience. Eur Radiol.

[22] Tekkis PP, Senagore AJ, Delaney CP, Fazio VW (2005). Evaluation of the learning curve in laparoscopic colorectal surgery: comparison of right-sided and left-sided resections. Ann Surg.

[23] Huang KH, Lan YT, Fang WL (2014). Comparison of the operative outcomes and learning curves between laparoscopic and robotic gastrectomy for gastric cancer. PLoS One.

[24] Heusch P, Kropil P, Buchbender C (2014). Radiation exposure of the radiologist's eye lens during CT-guided interventions. Acta Radiol.

[25] Rathmann N, Haeusler U, Diezler P (2015). Evaluation of radiation exposure of medical staff during CT-guided interventions. J Am Coll Radiol.

[26] Elsholtz FHJ, Vahldiek JL, Wyschkon S (2020). Radiation exposure of radiologists during different types of CT-guided interventions: an evaluation using dosimeters placed above and under lead protection. Acta Radiol.

[27] Nawfel RD, Judy PF, Silverman SG, Hooton S, Tuncali K, Adams DF (2000). Patient and personnel exposure during CT fluoroscopy-guided interventional procedures. Radiology.

[28] Gupta S, Wallace MJ, Cardella JF (2010). Quality improvement guidelines for percutaneous needle biopsy. J Vasc Interv Radiol.

[29] Pradella M, Trumm C, Stieltjes B, Boll DT, Zech CJ, Huegli RW (2019). Impact factors for safety, success, duration and radiation exposure in CT-guided interventions. Br J Radiol.

