# Correction to: Impact of interventionalist’s experience and gender on radiation dose and procedural time in CT-guided interventions—a retrospective analysis of 4380 cases over 10 years

**DOI:** 10.1007/s00330-021-08138-8

**Published:** 2021-07-12

**Authors:** Dorothea Theilig, Anna Mayerhofer, David Petschelt, Aboelyazid Elkilany, Bernd Hamm, Bernhard Gebauer, Dominik Geisel

**Affiliations:** Charité – Universitätsmedizin Berlin, corporate member of Freie Universität Berlin, Humboldt-Universität zu Berlin, Department of Diagnostic and Interventional Radiology, Augustenburger Platz 1, 13353 Berlin, Germany

**Correction to: European Radiology (2021) 31:569–579**

10.1007/s00330-020-07185-x

The article “Impact of interventionalist’s experience and gender on radiation dose and procedural time in CT-guided interventions—a retrospective analysis of 4380 cases over 10 years“, written by Theilig, D., Mayerhofer, A., Petschelt, D., Elkilany A., Hamm, B., Gebauer, B. and Geisel, D., was originally published Online First without Open Access. After publication in volume 31, issue 2, page 569–579 the author decided to opt for Open Choice and to make the article an Open Access publication. Therefore, the copyright of the article has been changed to © The Author(s) 2020 and the article is forthwith distributed under the terms of the Creative Commons Attribution 4.0 International License, which permits use, sharing, adaptation, distribution and reproduction in any medium or format, as long as you give appropriate credit to the original author(s) and the source, provide a link to the Creative Commons license, and indicate if changes were made. The images or other third party material in this article are included in the article’s Creative Commons license, unless indicated otherwise in a credit line to the material. If material is not included in the article’s Creative Commons license and your intended use is not permitted by statutory regulation or exceeds the permitted use, you will need to obtain permission directly from the copyright holder. To view a copy of this license, visit http://creativecommons.org/licenses/by/4.0/.

The original article has been corrected.

