# Supplementary material for: Impact of interventionalist’s experience and gender on radiation dose and procedural time in CT-guided interventions—a retrospective analysis of 4380 cases over 10 years
Source: Eur Radiol. 2020 Aug 26;31(2):569–79. doi: 10.1007/s00330-020-07185-x (PMC8263432; doi:10.1007/s00330-020-07185-x)
Supplement: Supplementary file 1 — (DOCX 36243 kb) [file 330_2020_7185_MOESM1_ESM.docx]

**Standard approach to intervention types**

For all CT-guided interventions the shortest puncture pathway without traversing critical structures was chosen.

Basic principles additionally observed for pathway planning when performing lung biopsies were choosing an angle perpendicular to the pleura, not crossing a lung fissure, avoiding large bronchi or blood vessels and passing at the upper edge of ribs in order to minimize the likelihood of costal nerve or vessel injury.

With liver biopsies and abdominal drains, the approach varies considerable with target lesion location necessitating an out-of-plane puncture pathway compared to target lesions that can be reached with an in-plane puncture pathway. An in-plane puncture describes a puncture pathway that lies within the axial plane of the patient so that the entire pathway and target lesion can be visualized on less than three 5 mm CT slices. An out-of-plane puncture describes a puncture pathway in a direction involving the longitudinal axis of the patient so that the needle tip and what lies ahead of it on the way to the target lesion cannot be visualized on the same CT slice, which renders the intervention more difficult. For that reason, the puncture technique with regards to in-plane versus out-of-plane puncture pathway was documented and included into the analysis.

The following techniques and equipment were routinely used for the different intervention types, slight variations in material choice occasionally occurred if the interventionist deemed it advantageous:

1) Periradicular therapy (PRT):

a. Cervical spine: 22 Gauge spinal cannula, test injection with 1 ml iodine contrast medium (Accupaque® 240, GE Healthcare); therapeutic injection of 1 ml dexamethasone palmitate (Lipotalon®, Recordati Pharma) and bupivacaine hydrochloride (Carbostesin® 0.5%, Aspen Pharma).

b. Lumbar spine: 20 Gauge spinal cannula; test injection with 1 ml iodine contrast medium (Accupaque® 240, GE Healthcare); therapeutic injection of 1 ml triamcinolone acetonide (Triam 40®, Zentiva Pharma) and 2 ml bupivacaine hydrochloride (Carbostesin® 0.5%, Aspen Pharma).

2) Liver biopsy:

Coaxial technique using a 17 Gauge coaxial needle (Co-axial Introducer Needle, Argon Medical Devices) combined with an 18 Gauge cutting needle (Quick-Core® Biopsy Needle, Cook Medical) and taking 3 samples, each 2 cm in length.

3) Lung biopsy:

Coaxial technique using a 19 Gauge coaxial needle (Co-axial Introducer Needle, Argon Medical Devices) combined with a 20 Gauge cutting needle (Quick-Core® Biopsy Needle, Cook Medical) and taking 2 samples, each 2 cm in length.

4) Drain insertion into abdominal fluid collection:

Seldinger technique using an 18 Gauge trocar needle (Cook Medical), a 0,035’’ guidewire (Amplatz®, Boston Scientific) and a 12 French sump drainage tube (Cook Medical).

5) Drain insertion into pleural fluid collection:

Seldinger technique using an 18 Gauge trocar needle (Cook Medical), a 0,035’’ guidewire (Amplatz®, Boston Scientific) and an 8,5 French multipurpose drainage tube (Cook Medical).

Figure I:

Boxplot diagram of DLPs of liver biopsies successively performed by each interventionalist in clusters of 10 displayed for female, male, and all interventionalists.


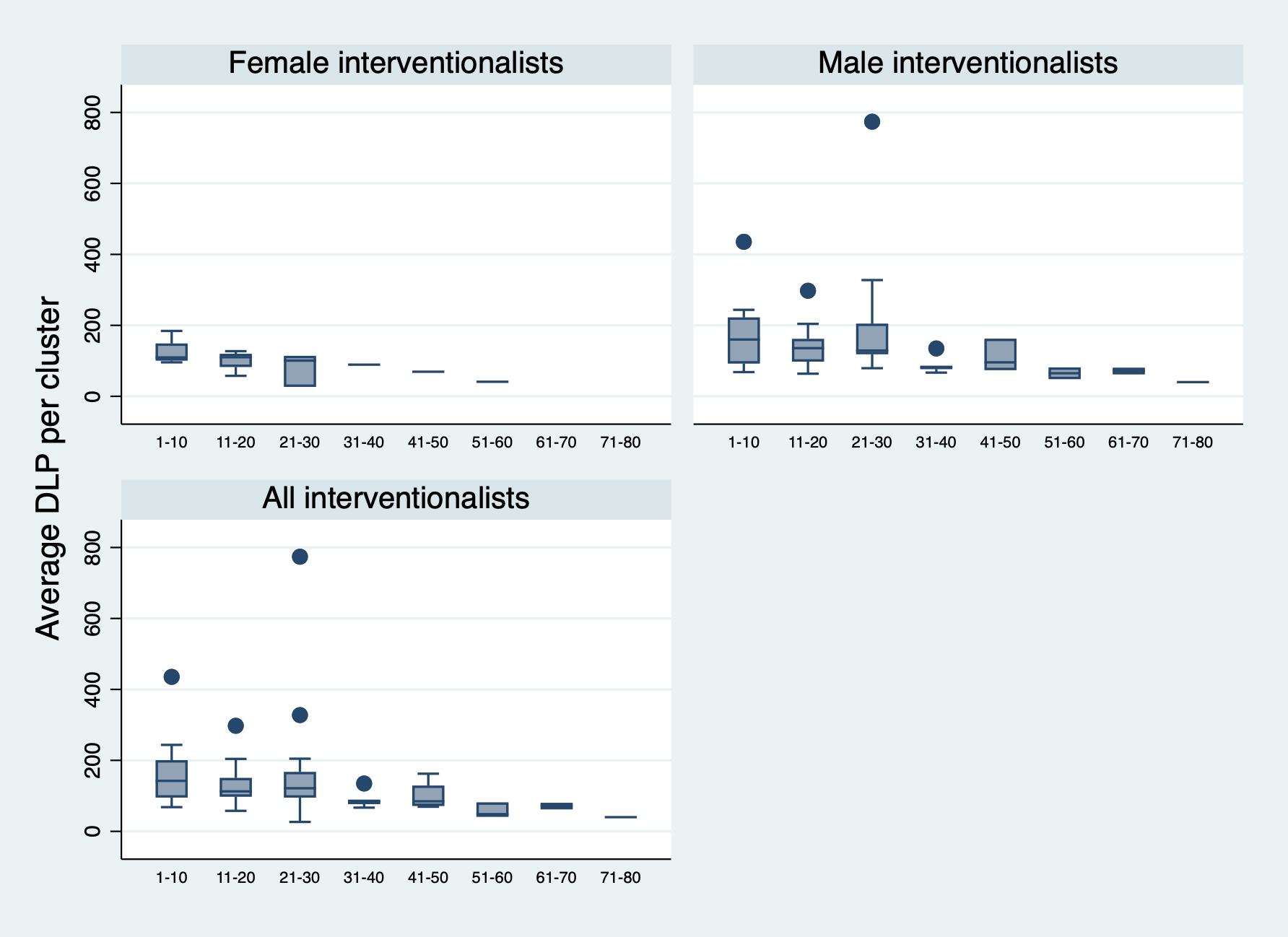


Figure II:

Boxplot diagram of DLPs of lung biopsies successively performed by each interventionalist in clusters of 10 displayed for female, male, and all interventionalists.


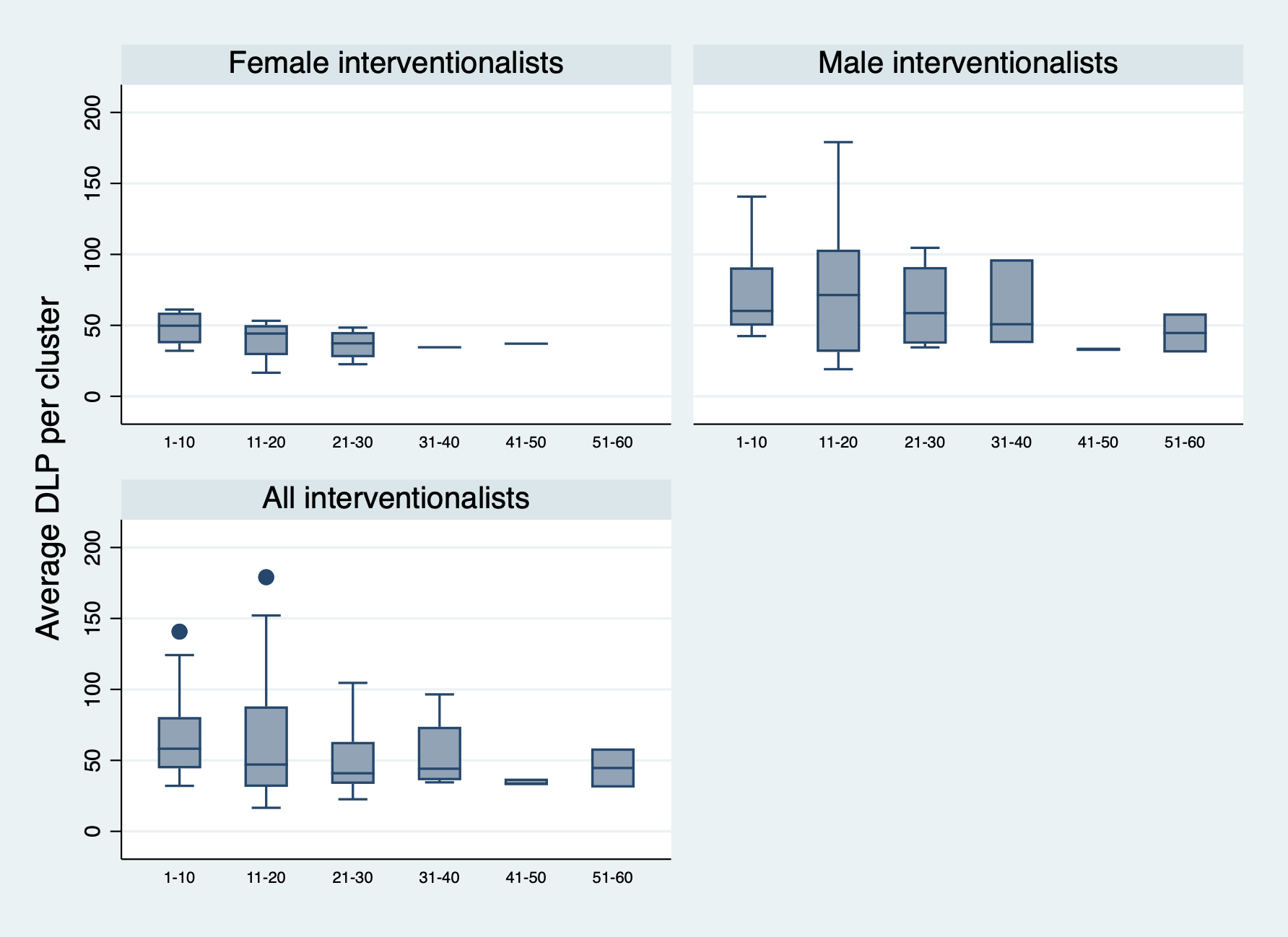


Figure III:

Boxplot diagram of DLPs of abdominal drains successively performed by each interventionalist in clusters of 10 displayed for female, male, and all interventionalists.


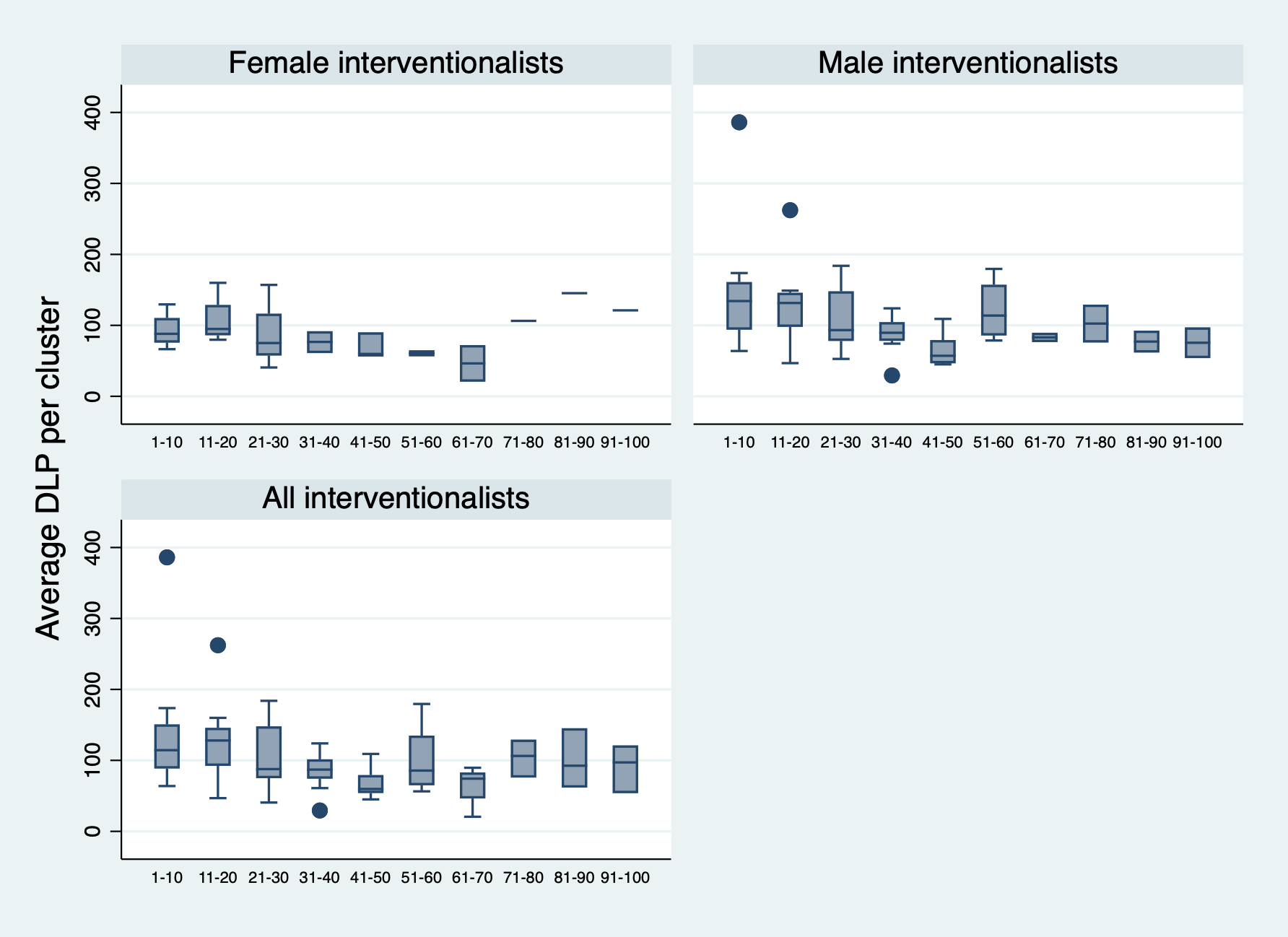


Figure IV:

Boxplot diagram of DLPs of chest drains successively performed by each interventionalist in clusters of 10 displayed for female, male, and all interventionalists.


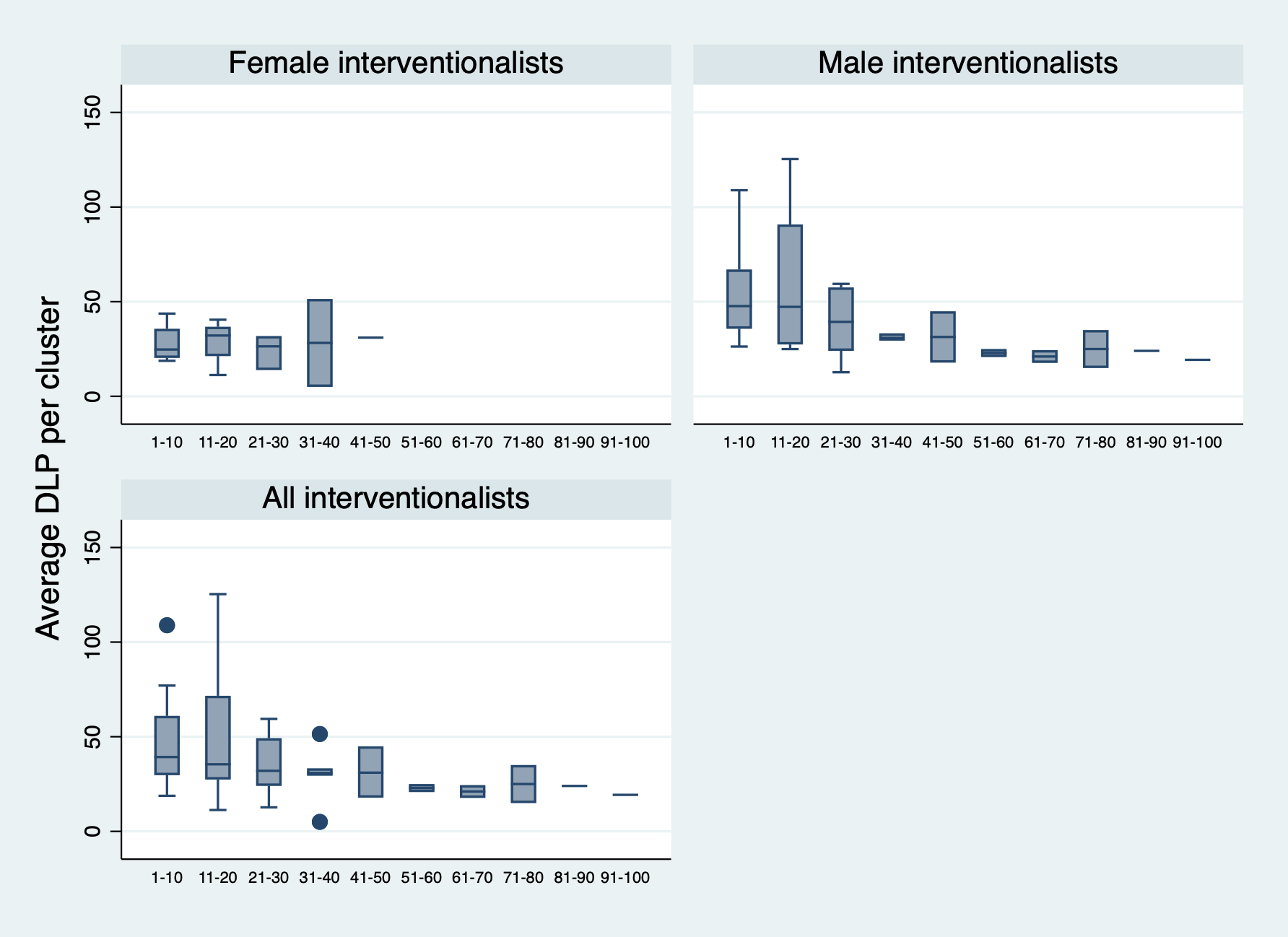


Table I:

Pairwise comparison of periradicular therapies successively performed by each interventionalist in clusters of 10.

| Clusters of 10 compared | Std.Err. | t | P>t | [95%Conf. | Interval] |
| --- | --- | --- | --- | --- | --- |
| 11-20 vs 1-10 | 11.482 | -0.610 | 0.541 | -29.754 | 15.674 |
| 21-30 vs 1-10 | 11.482 | -1.380 | 0.169 | -38.608 | 6.821 |
| 31-40 vs 1-10 | 11.482 | -1.480 | 0.143 | -39.651 | 5.778 |
| 41-50 vs 1-10 | 11.482 | -2.170 | 0.032 | -47.620 | -2.191 |
| 51-60 vs 1-10 | 11.482 | -2.490 | 0.014 | -51.351 | -5.923 |
| 61-70 vs 1-10 | 11.692 | -2.670 | 0.009 | -54.329 | -8.071 |
| 71-80 vs 1-10 | 11.929 | -2.870 | 0.005 | -57.788 | -10.591 |
| 81-90 vs 1-10 | 12.200 | -2.910 | 0.004 | -59.590 | -11.319 |
| 91-100 vs 1-10 | 12.513 | -3.170 | 0.002 | -64.455 | -14.947 |
| 21-30 vs 11-20 | 11.666 | -0.760 | 0.449 | -31.931 | 14.224 |
| 31-40 vs 11-20 | 11.666 | -0.850 | 0.398 | -32.974 | 13.181 |
| 41-50 vs 11-20 | 11.666 | -1.530 | 0.128 | -40.943 | 5.212 |
| 51-60 vs 11-20 | 11.666 | -1.850 | 0.066 | -44.675 | 1.481 |
| 61-70 vs 11-20 | 11.872 | -2.040 | 0.044 | -47.646 | -0.674 |
| 71-80 vs 11-20 | 12.106 | -2.240 | 0.027 | -51.098 | -3.201 |
| 81-90 vs 11-20 | 12.373 | -2.300 | 0.023 | -52.892 | -3.937 |
| 91-100 vs 11-20 | 12.682 | -2.580 | 0.011 | -57.749 | -7.573 |
| 31-40 vs 21-30 | 11.666 | -0.090 | 0.929 | -24.121 | 22.034 |
| 41-50 vs 21-30 | 11.666 | -0.770 | 0.441 | -32.090 | 14.066 |
| 51-60 vs 21-30 | 11.666 | -1.090 | 0.277 | -35.822 | 10.334 |
| 61-70 vs 21-30 | 11.872 | -1.290 | 0.200 | -38.793 | 8.179 |
| 71-80 vs 21-30 | 12.106 | -1.510 | 0.133 | -42.245 | 5.653 |
| 81-90 vs 21-30 | 12.373 | -1.580 | 0.116 | -44.039 | 4.916 |
| 91-100 vs 21-30 | 12.682 | -1.880 | 0.063 | -48.896 | 1.281 |
| 41-50 vs 31-40 | 11.666 | -0.680 | 0.496 | -31.047 | 15.109 |
| 51-60 vs 31-40 | 11.666 | -1.000 | 0.318 | -34.778 | 11.377 |
| 61-70 vs 31-40 | 11.872 | -1.200 | 0.232 | -37.750 | 9.223 |
| 71-80 vs 31-40 | 12.106 | -1.430 | 0.156 | -41.202 | 6.696 |
| 81-90 vs 31-40 | 12.373 | -1.500 | 0.137 | -42.996 | 5.960 |
| 91-100 vs 31-40 | 12.682 | -1.790 | 0.075 | -47.852 | 2.324 |
| 51-60 vs 41-50 | 11.666 | -0.320 | 0.750 | -26.809 | 19.346 |
| 61-70 vs 41-50 | 11.872 | -0.530 | 0.597 | -29.781 | 17.192 |
| 71-80 vs 41-50 | 12.106 | -0.770 | 0.445 | -33.233 | 14.665 |
| 81-90 vs 41-50 | 12.373 | -0.850 | 0.395 | -35.027 | 13.929 |
| 91-100 vs 41-50 | 12.682 | -1.170 | 0.245 | -39.884 | 10.293 |
| 61-70 vs 51-60 | 11.872 | -0.220 | 0.829 | -26.049 | 20.923 |
| 71-80 vs 51-60 | 12.106 | -0.460 | 0.647 | -29.501 | 18.397 |
| 81-90 vs 51-60 | 12.373 | -0.550 | 0.583 | -31.295 | 17.660 |
| 91-100 vs 51-60 | 12.682 | -0.870 | 0.385 | -36.152 | 14.024 |
| 71-80 vs 61-70 | 12.305 | -0.240 | 0.808 | -27.332 | 21.353 |
| 81-90 vs 61-70 | 12.568 | -0.340 | 0.736 | -29.118 | 20.609 |
| 91-100 vs 61-70 | 12.872 | -0.660 | 0.510 | -33.965 | 16.964 |
| 81-90 vs 71-80 | 12.790 | -0.100 | 0.921 | -26.566 | 24.036 |
| 91-100 vs 71-80 | 13.088 | -0.420 | 0.674 | -31.403 | 20.380 |
| 91-100 vs 81-90 | 13.336 | -0.320 | 0.751 | -30.628 | 22.135 |

Table II:

Pairwise comparison of liver biopsies successively performed by each interventionalist in clusters of 10.

| Clusters of 10 compared | Std.Err. | t | P>t | [95%Conf. | Interval] |
| --- | --- | --- | --- | --- | --- |
| 11-20 vs 1-10 | 39.828 | -0.780 | 0.439 | -111.057 | 48.859 |
| 21-30 vs 1-10 | 42.320 | 0.560 | 0.577 | -61.220 | 108.700 |
| 31-40 vs 1-10 | 53.050 | -1.360 | 0.181 | -178.457 | 34.549 |
| 41-50 vs 1-10 | 61.949 | -0.980 | 0.331 | -185.175 | 63.562 |
| 51-60 vs 1-10 | 69.722 | -1.490 | 0.141 | -244.103 | 35.842 |
| 61-70 vs 1-10 | 83.114 | -1.080 | 0.285 | -256.720 | 76.996 |
| 71-80 vs 1-10 | 114.229 | -1.060 | 0.294 | -350.423 | 108.227 |
| 21-30 vs 11-20 | 42.920 | 1.280 | 0.207 | -31.326 | 141.004 |
| 31-40 vs 11-20 | 53.530 | -0.760 | 0.449 | -148.321 | 66.612 |
| 41-50 vs 11-20 | 62.361 | -0.480 | 0.636 | -154.902 | 95.488 |
| 51-60 vs 11-20 | 70.088 | -1.040 | 0.302 | -213.738 | 67.676 |
| 61-70 vs 11-20 | 83.421 | -0.700 | 0.484 | -226.238 | 108.712 |
| 71-80 vs 11-20 | 114.453 | -0.790 | 0.435 | -319.772 | 139.775 |
| 31-40 vs 21-30 | 55.409 | -1.730 | 0.090 | -206.932 | 15.545 |
| 41-50 vs 21-30 | 63.981 | -1.320 | 0.192 | -212.994 | 43.901 |
| 51-60 vs 21-30 | 71.533 | -1.790 | 0.080 | -271.479 | 15.738 |
| 61-70 vs 21-30 | 84.639 | -1.340 | 0.185 | -283.522 | 56.318 |
| 71-80 vs 21-30 | 115.344 | -1.260 | 0.215 | -376.400 | 86.724 |
| 41-50 vs 31-40 | 71.533 | 0.160 | 0.877 | -132.461 | 154.756 |
| 51-60 vs 31-40 | 78.361 | -0.410 | 0.683 | -189.492 | 125.138 |
| 61-70 vs 31-40 | 90.483 | -0.200 | 0.844 | -199.561 | 163.743 |
| 71-80 vs 31-40 | 119.698 | -0.410 | 0.683 | -289.447 | 191.159 |
| 51-60 vs 41-50 | 84.639 | -0.510 | 0.611 | -213.244 | 126.596 |
| 61-70 vs 41-50 | 95.972 | -0.300 | 0.763 | -221.727 | 163.615 |
| 71-80 vs 41-50 | 123.899 | -0.490 | 0.629 | -309.029 | 188.446 |
| 61-70 vs 51-60 | 101.163 | 0.140 | 0.888 | -188.825 | 217.361 |
| 71-80 vs 51-60 | 127.962 | -0.130 | 0.895 | -273.863 | 239.927 |
| 71-80 vs 61-70 | 135.724 | -0.230 | 0.819 | -303.714 | 241.242 |

Table III:

Pairwise comparison of lung biopsies successively performed by each interventionalist in clusters of 10.

| Clusters of 10 compared | Std.Err. | t | P>t | [95%Conf. | Interval] |
| --- | --- | --- | --- | --- | --- |
| 11-20 vs 1-10 | 12.840 | -0.080 | 0.935 | -26.970 | 24.853 |
| 21-30 vs 1-10 | 15.470 | -1.090 | 0.282 | -48.066 | 14.371 |
| 31-40 vs 1-10 | 19.971 | -0.610 | 0.542 | -52.575 | 28.032 |
| 41-50 vs 1-10 | 22.477 | -1.450 | 0.154 | -78.024 | 12.696 |
| 51-60 vs 1-10 | 26.794 | -0.840 | 0.407 | -76.536 | 31.609 |
| 21-30 vs 11-20 | 15.640 | -1.010 | 0.319 | -47.352 | 15.775 |
| 31-40 vs 11-20 | 20.104 | -0.560 | 0.580 | -51.783 | 29.359 |
| 41-50 vs 11-20 | 22.595 | -1.400 | 0.169 | -77.203 | 13.993 |
| 51-60 vs 11-20 | 26.893 | -0.800 | 0.431 | -75.677 | 32.868 |
| 31-40 vs 21-30 | 21.877 | 0.210 | 0.835 | -39.574 | 48.726 |
| 41-50 vs 21-30 | 24.186 | -0.650 | 0.517 | -64.626 | 32.993 |
| 51-60 vs 21-30 | 28.243 | -0.200 | 0.843 | -62.614 | 51.381 |
| 41-50 vs 31-40 | 27.286 | -0.750 | 0.459 | -75.457 | 34.672 |
| 51-60 vs 31-40 | 30.939 | -0.330 | 0.743 | -72.630 | 52.245 |
| 51-60 vs 41-50 | 32.613 | 0.310 | 0.756 | -55.615 | 76.015 |

Table IV:

Pairwise comparison of abdominal drains successively performed by each interventionalist in clusters of 10.

| Clusters of 10 compared | Std.Err. | t | P>t | [95%Conf. | Interval] |
| --- | --- | --- | --- | --- | --- |
| 11-20 vs 1-10 | 17.727 | -0.540 | 0.591 | -44.898 | 25.745 |
| 21-30 vs 1-10 | 18.051 | -1.670 | 0.099 | -66.112 | 5.822 |
| 31-40 vs 1-10 | 19.319 | -2.460 | 0.016 | -85.961 | -8.973 |
| 41-50 vs 1-10 | 20.552 | -3.170 | 0.002 | -106.029 | -24.130 |
| 51-60 vs 1-10 | 23.612 | -1.300 | 0.199 | -77.629 | 16.466 |
| 61-70 vs 1-10 | 27.573 | -2.430 | 0.017 | -122.007 | -12.127 |
| 71-80 vs 1-10 | 31.032 | -0.900 | 0.370 | -89.826 | 33.840 |
| 81-90 vs 1-10 | 31.032 | -1.030 | 0.307 | -93.735 | 29.931 |
| 91-100 vs 1-10 | 31.032 | -1.320 | 0.190 | -102.890 | 20.776 |
| 21-30 vs 11-20 | 18.329 | -1.120 | 0.265 | -57.090 | 15.953 |
| 31-40 vs 11-20 | 19.579 | -1.940 | 0.057 | -76.904 | 1.122 |
| 41-50 vs 11-20 | 20.797 | -2.670 | 0.009 | -96.941 | -14.065 |
| 51-60 vs 11-20 | 23.826 | -0.880 | 0.381 | -68.478 | 26.469 |
| 61-70 vs 11-20 | 27.756 | -2.070 | 0.042 | -112.796 | -2.186 |
| 71-80 vs 11-20 | 31.195 | -0.590 | 0.557 | -80.574 | 43.741 |
| 81-90 vs 11-20 | 31.195 | -0.720 | 0.476 | -84.483 | 39.832 |
| 91-100 vs 11-20 | 31.195 | -1.010 | 0.316 | -93.638 | 30.677 |
| 31-40 vs 21-30 | 19.873 | -0.870 | 0.386 | -56.920 | 22.276 |
| 41-50 vs 21-30 | 21.073 | -1.660 | 0.102 | -76.924 | 7.055 |
| 51-60 vs 21-30 | 24.067 | -0.020 | 0.986 | -48.392 | 47.519 |
| 61-70 vs 21-30 | 27.964 | -1.320 | 0.191 | -92.641 | 18.797 |
| 71-80 vs 21-30 | 31.380 | 0.070 | 0.946 | -60.374 | 64.678 |
| 81-90 vs 21-30 | 31.380 | -0.060 | 0.956 | -64.283 | 60.769 |
| 91-100 vs 21-30 | 31.380 | -0.350 | 0.729 | -73.438 | 51.614 |
| 41-50 vs 31-40 | 22.169 | -0.790 | 0.429 | -61.786 | 26.561 |
| 51-60 vs 31-40 | 25.033 | 0.670 | 0.502 | -32.993 | 66.765 |
| 61-70 vs 31-40 | 28.799 | -0.680 | 0.498 | -76.983 | 37.783 |
| 71-80 vs 31-40 | 32.126 | 0.610 | 0.546 | -44.539 | 83.487 |
| 81-90 vs 31-40 | 32.126 | 0.480 | 0.629 | -48.448 | 79.578 |
| 91-100 vs 31-40 | 32.126 | 0.200 | 0.842 | -57.603 | 70.423 |
| 51-60 vs 41-50 | 25.996 | 1.330 | 0.189 | -17.300 | 86.296 |
| 61-70 vs 41-50 | 29.640 | -0.070 | 0.947 | -61.046 | 57.071 |
| 71-80 vs 41-50 | 32.882 | 1.130 | 0.263 | -28.433 | 102.606 |
| 81-90 vs 41-50 | 32.882 | 1.010 | 0.316 | -32.342 | 98.697 |
| 91-100 vs 41-50 | 32.882 | 0.730 | 0.467 | -41.497 | 89.542 |
| 61-70 vs 51-60 | 31.838 | -1.150 | 0.255 | -99.925 | 26.953 |
| 71-80 vs 51-60 | 34.877 | 0.070 | 0.941 | -66.906 | 72.082 |
| 81-90 vs 51-60 | 34.877 | -0.040 | 0.970 | -70.815 | 68.173 |
| 91-100 vs 51-60 | 34.877 | -0.300 | 0.765 | -79.970 | 59.018 |
| 71-80 vs 61-70 | 37.672 | 1.040 | 0.303 | -35.988 | 114.137 |
| 81-90 vs 61-70 | 37.672 | 0.930 | 0.354 | -39.897 | 110.228 |
| 91-100 vs 61-70 | 37.672 | 0.690 | 0.492 | -49.052 | 101.072 |
| 81-90 vs 71-80 | 40.273 | -0.100 | 0.923 | -84.154 | 76.336 |
| 91-100 vs 71-80 | 40.273 | -0.320 | 0.747 | -93.309 | 67.180 |
| 91-100 vs 81-90 | 40.273 | -0.230 | 0.821 | -89.400 | 71.090 |

Table V:

Pairwise comparison of chest drains successively performed by each interventionalist in clusters of 10.

| Clusters of 10 compared | Std.Err. | t | P>t | [95%Conf. | Interval] |
| --- | --- | --- | --- | --- | --- |
| 11-20 vs 1-10 | 8.597 | 0.230 | 0.818 | -15.307 | 19.281 |
| 21-30 vs 1-10 | 9.642 | -1.300 | 0.201 | -31.910 | 6.885 |
| 31-40 vs 1-10 | 12.255 | -1.400 | 0.168 | -41.816 | 7.492 |
| 41-50 vs 1-10 | 15.049 | -1.060 | 0.296 | -46.192 | 14.357 |
| 51-60 vs 1-10 | 17.939 | -1.350 | 0.182 | -60.386 | 11.793 |
| 61-70 vs 1-10 | 17.939 | -1.460 | 0.152 | -62.208 | 9.971 |
| 71-80 vs 1-10 | 17.939 | -1.240 | 0.223 | -58.261 | 13.918 |
| 81-90 vs 1-10 | 24.655 | -0.940 | 0.352 | -72.762 | 26.439 |
| 91-100 vs 1-10 | 24.655 | -1.130 | 0.264 | -77.487 | 21.714 |
| 21-30 vs 11-20 | 9.765 | -1.480 | 0.144 | -34.144 | 5.145 |
| 31-40 vs 11-20 | 12.352 | -1.550 | 0.128 | -43.997 | 5.700 |
| 41-50 vs 11-20 | 15.128 | -1.180 | 0.243 | -48.338 | 12.529 |
| 51-60 vs 11-20 | 18.006 | -1.460 | 0.151 | -62.506 | 9.940 |
| 61-70 vs 11-20 | 18.006 | -1.560 | 0.125 | -64.328 | 8.118 |
| 71-80 vs 11-20 | 18.006 | -1.340 | 0.186 | -60.381 | 12.065 |
| 81-90 vs 11-20 | 24.704 | -1.020 | 0.314 | -74.846 | 24.549 |
| 91-100 vs 11-20 | 24.704 | -1.210 | 0.233 | -79.571 | 19.824 |
| 31-40 vs 21-30 | 13.101 | -0.350 | 0.724 | -31.005 | 21.707 |
| 41-50 vs 21-30 | 15.746 | -0.220 | 0.830 | -35.081 | 28.271 |
| 51-60 vs 21-30 | 18.528 | -0.640 | 0.528 | -49.056 | 25.490 |
| 61-70 vs 21-30 | 18.528 | -0.730 | 0.466 | -50.879 | 23.667 |
| 71-80 vs 21-30 | 18.528 | -0.520 | 0.605 | -46.931 | 27.615 |
| 81-90 vs 21-30 | 25.087 | -0.420 | 0.673 | -61.117 | 39.819 |
| 91-100 vs 21-30 | 25.087 | -0.610 | 0.543 | -65.842 | 35.094 |
| 41-50 vs 31-40 | 17.468 | 0.070 | 0.944 | -33.897 | 36.386 |
| 51-60 vs 31-40 | 20.012 | -0.360 | 0.723 | -47.394 | 33.125 |
| 61-70 vs 31-40 | 20.012 | -0.450 | 0.657 | -49.217 | 31.303 |
| 71-80 vs 31-40 | 20.012 | -0.250 | 0.803 | -45.269 | 35.250 |
| 81-90 vs 31-40 | 26.202 | -0.230 | 0.820 | -58.712 | 46.712 |
| 91-100 vs 31-40 | 26.202 | -0.410 | 0.684 | -63.437 | 41.987 |
| 51-60 vs 41-50 | 21.835 | -0.380 | 0.703 | -52.305 | 35.548 |
| 61-70 vs 41-50 | 21.835 | -0.470 | 0.643 | -54.128 | 33.726 |
| 71-80 vs 41-50 | 21.835 | -0.290 | 0.776 | -50.180 | 37.673 |
| 81-90 vs 41-50 | 27.620 | -0.260 | 0.794 | -62.808 | 48.320 |
| 91-100 vs 41-50 | 27.620 | -0.430 | 0.667 | -67.533 | 43.595 |
| 61-70 vs 51-60 | 23.919 | -0.080 | 0.940 | -49.942 | 46.297 |
| 71-80 vs 51-60 | 23.919 | 0.090 | 0.930 | -45.994 | 50.244 |
| 81-90 vs 51-60 | 29.295 | 0.040 | 0.969 | -57.800 | 60.069 |
| 91-100 vs 51-60 | 29.295 | -0.120 | 0.903 | -62.525 | 55.343 |
| 71-80 vs 61-70 | 23.919 | 0.170 | 0.870 | -44.172 | 52.067 |
| 81-90 vs 61-70 | 29.295 | 0.100 | 0.920 | -55.977 | 61.891 |
| 91-100 vs 61-70 | 29.295 | -0.060 | 0.952 | -60.702 | 57.166 |
| 81-90 vs 71-80 | 29.295 | -0.030 | 0.973 | -59.925 | 57.944 |
| 91-100 vs 71-80 | 29.295 | -0.200 | 0.846 | -64.650 | 53.218 |
| 91-100 vs 81-90 | 33.827 | -0.140 | 0.890 | -72.776 | 63.326 |
